# Primary central nervous system lymphoma after heart transplantation: A case report and literature review

**DOI:** 10.1097/MD.0000000000021844

**Published:** 2020-08-28

**Authors:** Fang Zhu, Qiuhui Li, Tao Liu, Yin Xiao, Huaxiong Pan, Xinxiu Liu, Gang Wu, Liling Zhang

**Affiliations:** a Cancer Center; b Department of Pathology, Union Hospital, Tongji Medical College, Huazhong University of Science and Technology, Wuhan, China.

**Keywords:** heart transplantation, primary central nervous system lymphoma, treatment

## Abstract

**Rationale:**

The heart transplantation is the most important treatment for patients with end-stage severe heart disease who failed to conventional therapy. Post-transplant lymphoproliferative disorder is the second most common malignancy in heart transplant recipients. However, primary central nervous system lymphoma (PCNSL) after heart transplantation is an extremely rare condition.

**Patients concerns:**

This report described a 53-year-old male who was diagnosed as PCNSL 17 months after heart transplantation.

**Diagnoses:**

The patient was admitted to the local hospital presenting with dizziness, headache, and reduced left-sided power and sensation for 1 week. He had a medical history of heart transplantation because of the dilated cardiomyopathy 17 months ago and had a 17-month history of immunosuppressive therapy with tacrolimus. A computed tomography scan of the brain revealed a bulky mass in the right temporal lobe. The emergency intracranial mass resection and cerebral decompression were performed in our hospital. The histopathology of the brain lesions showed diffuse large B-cell lymphoma. A further ^18^FDG positron emission tomography-computed tomography scan of the whole body showed no significantly increased metabolic activity in other regions. The final diagnosis of this patient was PCNSL after heart transplantation.

**Interventions:**

Given the poor health condition, with the patient's consent, the whole brain radiotherapy was performed with supportive care.

**Outcomes:**

The disease deteriorated rapidly during the period of receiving radiotherapy, and he died within 2 months from the diagnosis.

**Lessons:**

PCNSL after heart transplantation is an extremely rare phenomenon with extremely poor prognosis. We should pay close attention to the heart recipients, especially when the patients present with neurological symptoms and signs. The available treatment options for PCNS-post-transplant lymphoproliferative disorder include the reduction of immunosuppressive drugs, immune-chemotherapy, operation, radiotherapy. However, individual treatments for heart transplant recipients with PCNSL should be based on the performance status and tolerance to treatment, combined with the doctor's experience and supportive care.

## 1 Introduction

The heart transplantation is the most important treatment for patients with end-stage severe heart disease who failed to conventional therapy.^[1]^ In addition to multidrug regimens to manage the cardiac disease-related comorbidities, the heart transplant recipients must take a life-long immunosuppressive therapy to maintain long-term graft function in the post-transplant period. The incidence rate of malignancy in heart transplant recipients was reported ranging from 3% to 30%, which was significantly higher than that in the general population.^[2–5]^ The long-term intensive immunosuppression plays a critical role in the development of malignancy in heart transplant recipients.

Post-transplant lymphoproliferative disorder (PTLD) is a group of clinically and pathologically heterogeneous lymphoid disorders ranging from indolent polyclonal proliferation to aggressive lymphomas, and which is one of the most common malignancies in solid organ transplant (SOT) recipients.^[6]^ Diffuse large B cell lymphoma (DLBCL) is one of the most common pathologies of PTLD. Primary central nervous system lymphoma (PCNSL) is defined as lymphoma involving the brain, meninges, eyes, or spinal cord without any other systemic organ involvement. It is a rare malignancy with a poor prognosis and high mortality even in immunocompetent patients.

The primary central nervous system posttransplantation lymphoproliferative disorder (PCNS-PTLD) in heart transplantation recipients is an extremely rare condition with high mortality. To the best of knowledge, less than 50 cases have been reported in English literature. The treatment for this rare condition remains a challenge for physicians. Here we report a rare case of PCNSL after heart transplantation and have a literature review.

## 2 Case presentation

**Figure 1:**
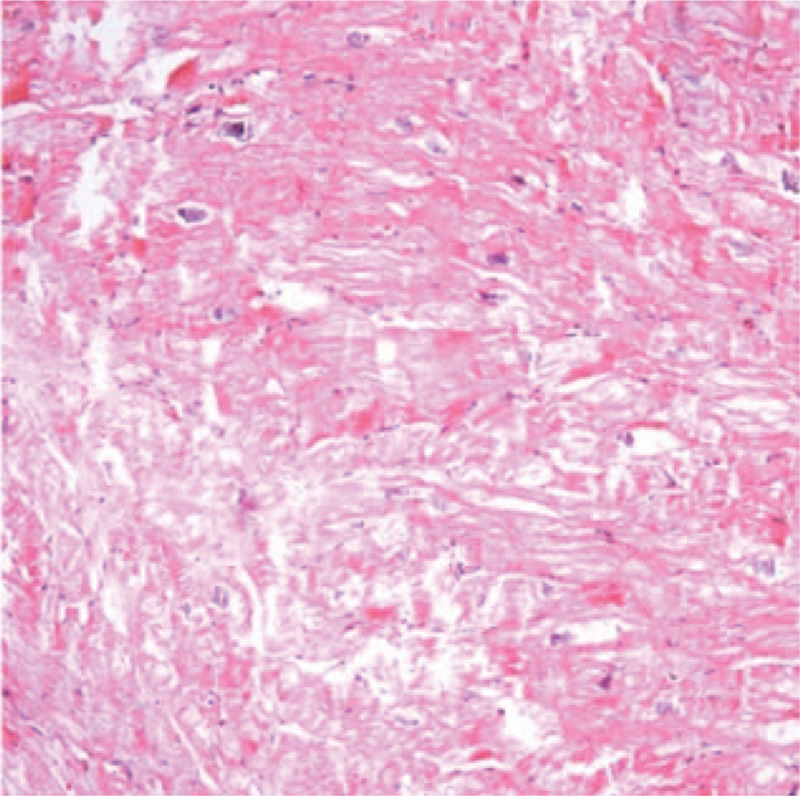
Hematoxylin-eosin staining confirmed dilated cardiomyopathy. (Original magnification ×200).

**Figure 2:**
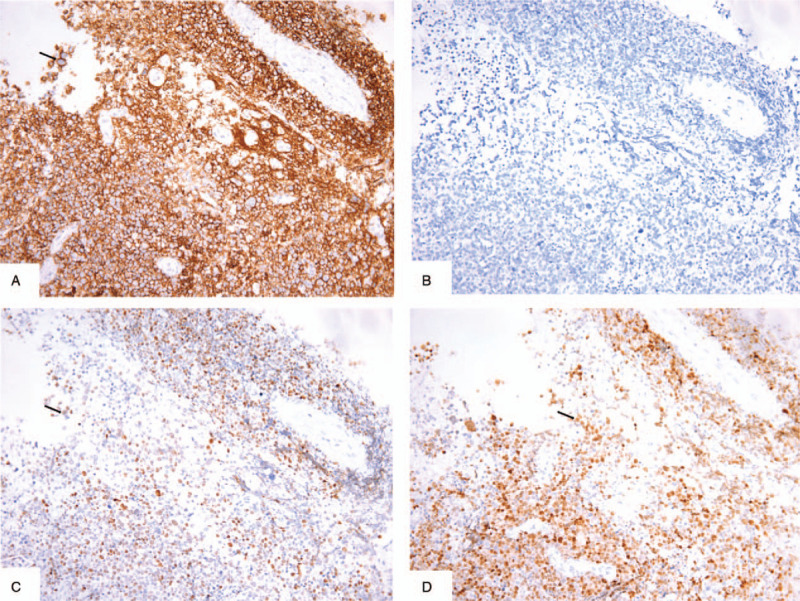
Pathology features of primary central nervous system posttransplant lymphoproliferative disorder. (A) Immunohistochemistry staining showed that the tumor cells (arrow) were CD20-positive, indicating a B-cell phenotype (original magnification ×200). (B) Immunohistochemistry staining showed that the tumor cells were CD10-negative (original magnification ×200). (C) Immunohistochemistry staining showed that the tumor cells (arrow) were MUM-1-positive (original magnification ×200). (D) Immunohistochemistry staining showed that the tumor cells (arrow) were BCL-6-positive (original magnification ×200).

**Figure 3:**
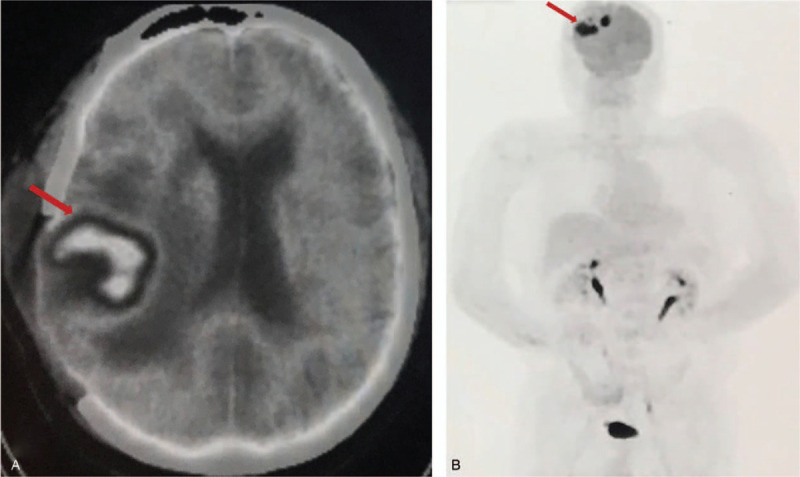
The ^18^FDG PET-CT scan showed significant FDG uptake in the right frontal and parietal lobes (arrow) combined brain edema (A) and no significantly increased metabolic activity in other regions (B). PET-CT =positron emission tomography-computed tomography.

A 53-year-old male was admitted to the local hospital presenting with dizziness, headache, and reduced left-sided power and sensation for 1 week. He had a medical history of heart transplantation because of the dilated cardiomyopathy confirmed by pathological examination (Fig. 1) 17 months ago, and had a 17-month history of immunosuppressive therapy with tacrolimus, without a personal or family medical history of a malignant neoplasm. He had no fever, night sweats, weight loss or any preceding symptoms. A computed tomography scan of the brain revealed a bulky mass in the right frontal and parietal lobes. The emergency intracranial mass resection and cerebral decompression were performed in the department of cerebral surgery of our hospital. The histological and immunohistochemical studies of the brain lesions identified diffuse large B-cell lymphoma (Fig. 2). The tumor cells were positive staining for CD20, LCA, Vimentin, PAX5, MUM-1, Bcl-6, Bcl-2, negative staining for GFAP, CD10, S-100, CD3, CyclinD1, CD34, and exhibited a high proliferation index as illustrated by Ki-67 staining (80% positive). The Epstein-Barr virus-encoded RNA was negative. The symptoms including dizziness, headache, and reduced left-sided power and sensation regressed after the operation. Then he went to the department of lymphoma to continue the specialized treatment with the Eastern Cooperative Oncology Group performance status score of 2. His complete blood count and lactate dehydrogenase were normal. A further ^18^FDG positron emission tomography-computed tomography scan of the whole body showed significant FDG uptake in the right frontal and parietal lobes combined brain edema and no significantly increased metabolic activity in other regions (Fig. 3). Bone marrow biopsy showed no evidence of lymphoma infiltration. The final diagnosis of this patient was PCNSL after heart transplantation. He refused to receive systemic chemotherapy. Given the poor health condition, with the patient's consent, the whole brain radiotherapy with the planning total dose of 30 Gy/15 f was performed. However, the disease deteriorated rapidly during the period of receiving radiotherapy, and that resulted in the termination of radiotherapy with a total dose of 24 Gy/12 f. He died within 2 months from the diagnosis.

## 3 Discussion

The first successful human heart transplant was performed in Australia in 1967.^[7]^ Graft vasculopathy and posttransplant malignancy are the 2 major causes of death in heart transplant recipients.^[8]^ The risk of malignancy in heart transplant recipients is higher than that in other types of SOT recipients due to the greater intensitive immunosuppressive therapy and Epstein-Barr virus infection.^[9,10]^ According to the International Society of Heart and Lung Transplant, the cumulative prevalence of malignancy in heart transplant recipients at 1 year is 2.9% and at 10 years is 31.9%.^[8]^ PTLD is the second most common malignancy in heart transplant recipients.^[3]^ The incidence rate of PTLD is 1.5% to 11.4%,^[2,11]^ and the majority of PTLD cases occur within 1 year after heart transplantation.^[12]^

Central nervous system (CNS) involvement in PTLD was first reported in 1970 by Schneck in a patient with kidney transplantation.^[13]^ CNS involvement occurred in approximately 7% to 15% of PTLD cases, but the primary involvement of CNS is rare.^[14]^ The immunosuppressed individuals such as patients with human immunodeficiency virus infection have a higher risk of PCNSL. The incidence rate of PCNSL is higher in SOT recipients than that in the general population because of the long-term immunosuppressive therapy. The subtype classification of PCNS-PTLD is usually diffuse large B cell lymphoma. In Mahale P's study including 17 cancer registries from the United States transplant registry, 168 cases of PCNS-PTLD were diagnosed among the 288,029 transplants with the median follow-up time of 4.0 years (range 1.5–7.7 years). The incidence rate of PCNS-PTLD was 11.5 per 100,000 person-years. PCNS-PTLD occurred at a median time of 1.7 years after SOT, especially within the first 1.5 years. The median survival time of PCNS-PTLD was 1.1 years. Recipients with PCNSL had higher mortality compared to those with systemic non-Hodgkin lymphoma.^[15]^

The precise occurrence of PCNS-PTLD in heart transplant recipients remains unclear because of the rarity. PCNS-PTLD in heart and/or lung transplantation recipients was nearly 10.1 per 100,000 person-years in Mahale P's study.^[15]^ According to Gifford G's a single-center study of 1674 cases with heart and/or lung transplant, only 2 heart transplant recipients were diagnosed as PCNS-PTLD during the 28 years of follow-up, yielding a total prevalence of 0.12%.^[16]^ The precise characteristics and treatment of PCNS-PTLD in heart transplant recipients were reported only in 7 cases according to a comprehensive literature search from the electronic databases PubMed with the keywords of “primary central nervous system” and “heart transplantation,” and they were reviewed in Table 1.^[16–19]^

**Table 1:**
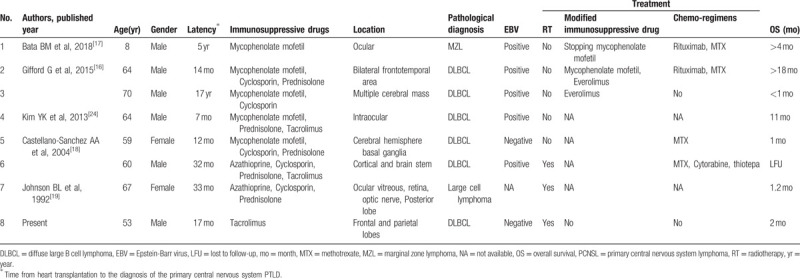
Clinical characteristics of reported cases of PCNS-PTLD after heart transplantation.

There is no consensus on the treatment of PCNS-PTLD in the patients after SOT because of the rarity and complexity. It is also difficult to conduct the prospective clinical trial to explore the evidence-based treatment strategy in PCNS-PTLD after SOT because of the small size population. The physicians usually explore the appropriate treatment according to the experience of PCNSL and PTLD patients. DLBCL is the most common pathology subtype of PCNSL, and it is sensitive to radiotherapy and chemotherapy. The first-line treatment strategy for PCNSL in immunocompetent patients mainly include surgery, high dose methotrexate-based combination chemotherapy, anti-CD20 monoclonal antibody rituximab, and the whole-brain radiotherapy. The treatment for PTLD includes reducing the dose of immunosuppressive drugs, systemic immune-chemotherapy, radiotherapy, and so on. However, the heart transplant recipients with PCNS-PTLD are usually with poor performance status or other cardiac abnormality, which severely limits the administration of anti-tumor therapy.

Though reducing the dose of immunosuppressive drugs or adjusting immunosuppressive drugs has been reported to be effective in some patients with low-grade systemic PTLD,^[20]^ the reduction of immunosuppressive drugs for heart transplant recipients may greatly increase the risk of graft failure, which is fetal comorbidity and may result in high mortality. Mahale P et al reported that there was a 3-fold elevated risk of graft failure/retransplantation in transplant recipients with PCNS-PTLD, which contributes to the high mortality.^[15]^

Rituximab, cyclophosphamide, adriamycin, vincristine, prednisone regimen chemotherapy is the standard treatment of DLBCL, which was confirmed to be effective in PTLD patients.^[21]^ However, the presence of the blood-brain barrier limits the application in PCNS-PTLD. The high dose methotrexate-based regimens have been identified to infiltrate to the blood-brain barrier and could be used to treat patients with PCNSL.^[22]^ The methotrexate- and/or cytarabine-based chemotherapy regimens were confirmed to be effective and well-tolerated in SOT recipients with PCNSLs.^[23]^ However, whether the heart transplant recipients could tolerate the high dose of methotrexate-based chemotherapy remains unclear because of the poor performance status or other heart disease-related comorbidities.

The purpose of surgery for PCNSL patients is mainly obtaining a histological diagnosis or cerebral decompression to relieve the uncomfortable symptoms due to intracranial lesions. It has a little therapeutic role in PCNS-PTLD patients after heart transplantation except in obtaining a biopsy or an emergency. The purpose of emergency intracranial mass resection and cerebral decompression in our case was to obtain a biopsy and to relieve the symptoms. Radiotherapy is an important treatment strategy especially in newly diagnosed PCNSL patients who are unable to tolerate systemic chemotherapy. The whole-brain radiotherapy or focal radiotherapy is optional according to the illness condition and the performance status of the patient. In our case, given the patient's poor performance, the whole-brain radiotherapy was performed. Unfortunately, the disease deteriorated quickly during the period of radiotherapy, he died with the survival time of only 2 months.

The occurrence of PCNS-PTLD after heart transplantation may increase because of the improvement of transplantation techniques. The available treatment options for PCNS-PTLD include a reduction the immunosuppressive drugs, immune-chemotherapy, surgery, radiotherapy. However, the poor performance status and other complex medical comorbidities are the major factors preventing the administration of standard treatment in heart transplant recipients with PCNS-PTLD. Therefore, heart transplant recipients with PCNSL had a poor prognosis and high mortality.

In conclusion, though PCNS-PTLD after heart transplantation is an extremely rare phenomenon with poor prognosis, we should pay close attention to the heart recipients, especially when the patients present with neurological symptoms and signs. The treatment for this rare condition is intractable. The available treatment options for PCNS-PTLD include reduction the immunosuppressive drugs, immune-chemotherapy, surgery, radiotherapy. However, individual treatments for this rare condition should be based on the performance status and tolerance to treatment, combined with the doctor's experience and supportive care.

## Acknowledgments

The authors would like to thank the patient and his families as well as all doctors in the department of pathology and Cancer Center, Union Hospital, Tongji Medical College, Huazhong University of Science and Technology.

## Author contributions

**Data curation:** Fang Zhu, Huaxiong Pan, Yin Xiao, Qiuhui Li, Tao Liu, Xinxiu Liu, Liling Zhang.

**Funding acquisition:** Liling Zhang.

**Methodology:** Liling Zhang, Gang Wu, Fang Zhu.

**Supervision:** Gang Wu, Liling Zhang.

**Validation:** Fang Zhu, Huaxiong Pan, Gang Wu, Liling Zhang.

**Writing – original draft:** Fang Zhu.

**Writing – review & editing:** Liling Zhang.
